# Vital Signs: Trends and Disparities in Infant Safe Sleep Practices — United States, 2009–2015

**DOI:** 10.15585/mmwr.mm6701e1

**Published:** 2018-01-12

**Authors:** Jennifer M. Bombard, Katherine Kortsmit, Lee Warner, Carrie K. Shapiro-Mendoza, Shanna Cox, Charlan D. Kroelinger, Sharyn E. Parks, Deborah L. Dee, Denise V. D’Angelo, Ruben A. Smith, Kim Burley, Brian Morrow, Christine K. Olson, Holly B. Shulman, Leslie Harrison, Carri Cottengim, Wanda D. Barfield

**Affiliations:** ^1^Division of Reproductive Health, National Center for Chronic Disease Prevention and Health Promotion, CDC; ^2^Oak Ridge Institute for Science and Education Fellowship.

**Introduction:** There have been dramatic improvements in reducing infant sleep-related deaths since the 1990s, when recommendations were introduced to place infants on their backs for sleep. However, there are still approximately 3,500 sleep-related deaths among infants each year in the United States, including those from sudden infant death syndrome, accidental suffocation and strangulation in bed, and unknown causes. Unsafe sleep practices, including placing infants in a nonsupine (on side or on stomach) sleep position, bed sharing, and using soft bedding in the sleep environment (e.g., blankets, pillows, and soft objects) are modifiable risk factors for sleep-related infant deaths.

**Methods:** CDC analyzed 2009–2015 Pregnancy Risk Assessment Monitoring System (PRAMS) data to describe infant sleep practices. PRAMS, a state-specific and population-based surveillance system, monitors self-reported behaviors and experiences before, during, and shortly after pregnancy among women with a recent live birth. CDC examined 2015 data on nonsupine sleep positioning, bed sharing, and soft bedding use by state and selected maternal characteristics, as well as linear trends in nonsupine sleep positioning from 2009 to 2015.

**Results:** In 2015, 21.6% of respondents from 32 states and New York City reported placing their infant in a nonsupine sleep position; this proportion ranged from 12.2% in Wisconsin to 33.8% in Louisiana. Infant nonsupine sleep positioning was highest among respondents who were non-Hispanic blacks. Nonsupine sleep positioning prevalence was higher among respondents aged <25 years compared with ≥25 years, those who had completed ≤12 years compared with >12 years of education, and those who participated in the Special Supplemental Nutrition Program for Women, Infants, and Children during pregnancy. Based on trend data from 15 states, placement of infants in a nonsupine sleep position decreased significantly from 27.2% in 2009 to 19.4% in 2015. In 2015, over half of respondents (61.4%) from 14 states reported bed sharing with their infant, and 38.5% from 13 states and New York City reported using any soft bedding, most commonly bumper pads and thick blankets.

**Conclusions and Implications for Public Health Practice:** Improved implementation of the safe sleep practices recommended by the American Academy of Pediatrics could help reduce sleep-related infant mortality. Evidence-based interventions could increase use of safe sleep practices, particularly within populations whose infants might be at higher risk for sleep-related deaths.

## Introduction

Approximately 3,500 sleep-related deaths among infants are reported each year in the United States, including those from sudden infant death syndrome (SIDS), accidental suffocation and strangulation in bed, and unknown causes (*1*). Significant sociodemographic and geographic disparities in sleep-related infant deaths exist (*2*,*3*). To reduce risk factors for sleep-related infant mortality, recommendations from the American Academy of Pediatrics (AAP) for safe sleep include 1) placing the infant in the supine sleep position (placing the infant on his or her back) on a firm sleep surface such as a mattress in a safety-approved crib or bassinet, 2) having infant and caregivers share a room, but not the same sleeping surface, and 3) avoiding the use of soft bedding (e.g., blankets, pillows, and soft objects) in the infant sleep environment (*4*). Additional recommendations to reduce the risk for sleep-related infant deaths include breastfeeding, providing routinely recommended immunizations, and avoiding prenatal and postnatal exposure to tobacco smoke, alcohol, and illicit drugs (*4*).

Although the individual effect of each recommendation on sleep-related infant mortality is unclear, sharp declines in SIDS and other sleep-related mortality in the 1990s have been attributed to an increase in safe sleep practices such as supine sleep. However, since the late 1990s declines in infant sleep-related deaths (*4*) and nonsupine sleep positioning (on side or stomach) (*5*) have been less pronounced. The rate of infant sleep-related deaths declined from 154.6 deaths per 100,000 live births in 1990 to 93.9 per 100,000 live births in 1999; in 2015, the rate of infant sleep-related deaths was 92.6 deaths per 100,000 live births (*6*). Previous research indicates implementation of safe sleep recommendations by infant caregivers remains suboptimal. In the Study of Attitudes and Factors Effecting Infant Care, which interviewed mothers 2–6 months postpartum during 2011–2014, 22% said they had placed their infant in a nonsupine sleep position (*7*), and 21% shared a bed with their infant at least once during the 2 weeks before being interviewed (*8*). In addition, in the National Infant Sleep Position Study, a household telephone survey that sampled nighttime caregivers during 2007–2010, more than half (54%) placed their infant to sleep with soft bedding during the 2 weeks before the interview (*9*).

CDC used data from the Pregnancy Risk Assessment Monitoring System (PRAMS) to examine the prevalence of unsafe infant sleep practices. Ongoing surveillance efforts can identify populations at risk for unsafe sleep practices and help evaluate policies and programs to improve safe sleep practices. Health care providers and state-based and community-based programs can identify barriers to safe sleep practices and provide culturally appropriate counseling and messaging to improve infant sleep practices.

## Methods

**Data source.** PRAMS (*10*) collects state-specific, population-based data on self-reported maternal behaviors and experiences before, during, and shortly after pregnancy. In each participating state, a stratified random sample of women with a recent live birth is selected from birth certificate files, and women are surveyed 2–6 months postpartum using a standardized protocol and questionnaire. PRAMS data for each site are weighted for sampling design, nonresponse, and noncoverage to produce a data set representative of the state’s birth population. PRAMS sites were included in this report if their weighted response rate was ≥65% for years 2009–2011, ≥60% for 2012–2014, and ≥55% for 2015.

PRAMS sites included the question, “In which position do you most often lay your baby down to sleep now?” (check one answer): “on side; on back; on stomach.” Respondents who selected “on side” or “on stomach” were classified as placing their infant in a nonsupine sleep position.* Analyses on nonsupine sleep positioning were conducted using 2015 data from 32 PRAMS states^†^ and New York City. To explore trends in nonsupine sleep position, CDC analyzed PRAMS data from 2009–2015 in 15 states.^§^ Analyses of bed sharing used 2015 data from 14 states^¶^ that included the optional question on their state-specific PRAMS survey: “How often does your new baby sleep in the same bed with you or anyone else?” Respondents who indicated “always,” “often,” “sometimes,” or “rarely” were classified as having bed shared and were compared with respondents who indicated “never.” Bed sharing was also categorized as: “rarely or sometimes,” and “often or always.” Analyses of soft bedding used 2015 data from 13 states** and New York City that included the following optional question on their state-specific survey: “Listed below are some things that describe how your new baby usually sleeps.” Respondents were asked to select “yes” or “no” for the following soft bedding items: “pillows,” “thick or plush blankets,” “bumper pads,” “stuffed toys” and “infant positioner.” Respondents who selected “yes” to one or more items were defined as using any soft bedding.

**Statistical analysis.** The weighted prevalence and 95% confidence intervals of unsafe sleep practices were calculated overall and by state for 2015. Chi-square tests and 95% confidence intervals^††^ were used to determine differences in unsafe sleep practices by maternal characteristic (i.e., race/ethnicity, age, education level, and participation in the Special Supplemental Nutrition Program for Women, Infants, and Children (WIC) program during pregnancy), gestational age at birth (i.e., preterm, <37 weeks’ gestation, compared with term, ≥37 weeks’ gestation) and any breastfeeding at 8 weeks postpartum. CDC tested for linear trends in nonsupine sleep position overall and by maternal characteristics and state, from 2009 to 2015, using logistic regression. Analyses accounted for the complex survey sampling design of PRAMS.

## Results

In 2015, the overall prevalence of nonsupine sleep positioning was 21.6%, ranging from 12.2% in Wisconsin to 33.8% in Louisiana (Table 1). Nonsupine sleep positioning varied by maternal characteristics, and was highest among respondents who were non-Hispanic blacks. Nonsupine sleep positioning prevalence was higher among respondents aged <25 years compared with ≥25 years and those who had completed ≤12 years compared with >12 years of education, and who were WIC participants. Among the 15 states examined during 2009–2015, nonsupine sleep positioning decreased significantly from 27.2% in 2009 to 19.4% in 2015 overall (p<0.001) (Supplementary Table https://stacks.cdc.gov/view/cdc/50001) and in 13 of 15 states (except for Maryland and Washington). Nonsupine sleep positioning decreased significantly among all age, education, WIC participation and most race/ethnicity groups except among respondents who were American Indians/Alaska Natives (Figure).^§§^

**TABLE 1:** Prevalence of nonsupine (on side or stomach) sleep positioning, by maternal characteristics, gestational age at birth, and breastfeeding at 8 weeks postpartum — Pregnancy Risk Assessment Monitoring System, 32 states and New York City, 2015

Characteristic	Nonsupine sleep positioning % (95% CI)*	Chi-square p-value
**Total**	**21.6 (20.9–22.4)**	**—**
**Maternal race/ethnicity**		<0.001
White, non-Hispanic	16.1 (15.3–16.9)	
Black, non-Hispanic	37.6 (35.8–39.3)	
Hispanic	26.5 (24.3–28.9)	
Asian or Pacific Islander, non-Hispanic	20.8 (18.2–23.6)	
American Indian or Alaska Native, non-Hispanic	19.8 (13.8–27.6)	
**Maternal age group (yrs)**		<0.001
<20	29.9 (26.4–33.5)	
20–24	27.9 (26.0–29.8)	
25–34	19.4 (18.6–20.3)	
≥35	18.5 (16.8–20.3)	
**Maternal education (yrs)**		<0.001
<12	27.9 (25.5–30.5)	
12	26.0 (24.3–27.7)	
>12	18.4 (17.6–19.2)	
**WIC participation during pregnancy**		<0.001
No	16.7 (15.9–17.6)	
Yes	28.0 (26.7–29.3)	
**Infant gestation (wks)**		0.240
Term (≥37)	21.5 (20.7–22.3)	
Preterm (<37)	22.9 (20.8–25.2)	
**Any breastfeeding at 8 wks**		<0.001
No	24.0 (22.7–25.4)	
Yes	20.4 (19.5–21.3)	
**State/City**		<0.001
Alabama	28.7 (25.7–32.0)	
Alaska	23.0 (20.1–26.2)	
Arkansas	29.3 (25.3–33.6)	
Colorado	12.3 (10.3–14.6)	
Connecticut	22.7 (19.7–26.1)	
Delaware	18.7 (16.1–21.5)	
Hawaii	18.5 (15.8–21.5)	
Illinois	19.1 (17.0–21.4)	
Iowa	14.2 (11.5–17.5)	
Louisiana	33.8 (30.9–36.8)	
Maryland	25.4 (22.7–28.3)	
Massachusetts	14.2 (12.1–16.5)	
Michigan	18.6 (16.3–21.1)	
Missouri	20.6 (17.9–23.5)	
Nebraska	15.9 (13.8–18.2)	
New Hampshire	13.1 (10.1–16.7)	
New Jersey	29.5 (26.8–32.3)	
New Mexico	21.7 (19.5–24.0)	
New York City	31.1 (28.6–33.8)	
New York (outside of New York City)	20.9 (17.6–24.6)	
Ohio	14.5 (12.1–17.3)	
Oklahoma	18.8 (16.0–21.9)	
Oregon	17.9 (15.1–21.2)	
Pennsylvania	16.0 (13.6–18.7)	
Tennessee	17.0 (14.1–20.4)	
Texas	28.8 (25.7–32.0)	
Utah	16.4 (14.1–18.9)	
Vermont	15.3 (13.0–18.0)	
Virginia	22.0 (18.2–26.2)	
Washington	17.5 (15.1–20.2)	
West Virginia	16.3 (13.7–19.3)	
Wisconsin	12.2 (9.8–15.1)	
Wyoming	12.5 (9.6–16.2)	

**Abbreviations:** CI = confidence interval; WIC = Special Supplemental Nutrition Program for Women, Infants, and Children.

* Weighted percentage.

**FIGURE:**
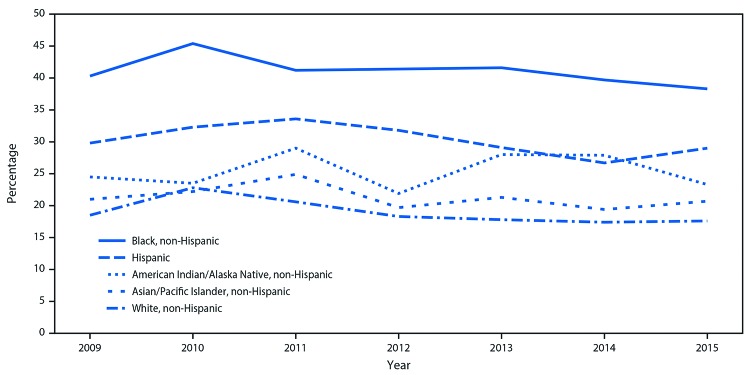
Trends in prevalence of nonsupine (on side or stomach) sleep positioning of infants, by mother’s race/ethnicity — 15 states,* Pregnancy Risk Assessment Monitoring System, 2009–2015 * Delaware, Hawaii, Illinois, Maryland, Massachusetts, Missouri, Nebraska, New Jersey, Oklahoma, Pennsylvania, Utah, Vermont, Washington, West Virginia and Wyoming.

In 2015, more than half (61.4%) of respondents reported any bed sharing with their infant, with 37.0% reporting “rarely or sometimes” and 24.4% responding “often or always” bed sharing (Table 2). Self-report of any bed sharing varied by state, ranging from 49.0% in West Virginia to 78.9% in Alaska. The prevalence of bed sharing varied by maternal characteristics, gestational age at birth, and breastfeeding at 8 weeks postpartum. Bed sharing prevalence was higher among respondents who were American Indians/Alaska Natives, non-Hispanic blacks, or Asians/Pacific Islanders compared with non-Hispanic whites or Hispanics, aged <25 years compared with ≥25 years, who had completed ≤12 years compared with >12 years of education, who were WIC participants, and who reported any breastfeeding at 8 weeks postpartum (Table 2).

**TABLE 2:** Prevalence of bed sharing, by maternal characteristics, gestational age at birth, and breastfeeding at 8 weeks postpartum — Pregnancy Risk Assessment Monitoring System, 14 states, 2015

Characteristic	Any*	Rarely or sometimes	Often or always	Never	Chi–square p–value
	% (95% CI)^†^	% (95% CI)^†^	% (95% CI)^†^	% (95% CI)^†^	Never versus Any
**Total**	**61.4 (59.9–62.8)**	**37.0 (35.6–38.5)**	**24.4 (23.1–25.7)**	**38.6 (37.2–40.1)**	**—**
**Maternal race/ethnicity**					<0.001
White, non-Hispanic	52.7 (50.9–54.4)	35.2 (33.5–37.0)	17.5 (16.1–18.9)	47.3 (45.6–49.1)	
Black, non-Hispanic	76.5 (74.2–78.7)	41.2 (38.5–43.9)	35.3 (32.7–38.0)	23.5 (21.3–25.8)	
Hispanic	66.7 (62.9–70.3)	38.0 (34.3–41.9)	28.7 (25.2–32.4)	33.3 (29.7–37.1)	
Asian or Pacific Islander, non-Hispanic	76.8 (72.0–80.9)	39.8 (34.7–45.2)	37.0 (31.8–42.4)	23.2 (19.1–28.0)	
American Indian or Alaska Native, non-Hispanic	83.9 (75.3–89.9)	27.8 (20.1–37.0)	56.1 (44.3–67.3)	16.1 (10.1–24.7)	
**Maternal age group (yrs)**					<0.001
<20	76.8 (71.1–81.7)	40.5 (34.3–47.2)	36.3 (30.0–43.1)	23.2 (18.3–28.9)	
20–24	68.5 (65.2–71.7)	40.5 (37.1–44.0)	28.0 (24.9–31.3)	31.5 (28.3–34.8)	
25–34	58.1 (56.3–59.9)	36.3 (34.5–38.2)	21.8 (20.3–23.4)	41.9 (40.1–43.7)	
≥35	57.1 (53.6–60.6)	33.5 (30.3–36.9)	23.6 (20.5–27.0)	42.9 (39.4–46.4)	
**Maternal education level (yrs)**					0.001
<12	65.2 (60.7–69.4)	34.4 (30.2–38.9)	30.8 (26.5–35.5)	34.8 (30.6–39.3)	
12	64.6 (61.5–67.5)	39.9 (36.8–42.9)	24.7 (22.1–27.6)	35.4 (32.5–38.5)	
>12	58.8 (57.1–60.5)	36.3 (34.6–38.0)	22.5 (21.1–24.0)	41.2 (39.5–42.9)	
**WIC participation during pregnancy**					<0.001
No	57.5 (55.7–59.3)	35.4 (33.7–37.2)	22.1 (20.5–23.7)	42.5 (40.7–44.3)	
Yes	66.2 (63.9–68.5)	39.0 (36.6–41.4)	27.2 (25.1–29.5)	33.8 (31.5–36.1)	
**Infant gestation (wks)**					0.023
Term (≥37)	61.8 (60.3–63.3)	37.0 (35.5–38.5)	24.8 (23.4–26.2)	38.2 (36.7–39.7)	
Preterm (<37)	56.4 (52.1–60.7)	37.5 (33.3–41.9)	18.9 (15.9–22.3)	43.6 (39.3–47.9)	
**Any breastfeeding at 8 wks**					<0.001
No	56.9 (54.3–59.4)	36.6 (34.0–39.1)	20.3 (18.3–22.5)	43.1 (40.6–45.7)	
Yes	63.8 (62.1–65.5)	37.4 (35.6–39.1)	26.4 (24.8–28.1)	36.2 (34.5–37.9)	
**State**					<0.001
Alaska	78.9 (75.7–81.7)	33.0 (29.7–36.4)	45.9 (42.4–49.4)	21.1 (18.3–24.3)	
Connecticut	52.9 (48.9–56.9)	33.8 (30.2–37.6)	19.1 (16.3–22.3)	47.1 (43.1–51.1)	
Delaware	52.8 (49.5–56.2)	34.4 (31.3–37.7)	18.4 (15.9–21.1)	47.2 (43.8–50.5)	
Louisiana	63.6 (60.5–66.7)	35.5 (32.5–38.7)	28.1 (25.4–31.0)	36.4 (33.3–39.5)	
Nebraska	54.4 (51.2–57.6)	35.2 (32.2–38.4)	19.2 (16.9–21.7)	45.6 (42.4–48.8)	
New Jersey	57.7 (54.6–60.8)	37.9 (34.9–41.1)	19.8 (17.5–22.3)	42.3 (39.2–45.4)	
Pennsylvania	50.9 (47.4–54.3)	37.4 (34.1–40.7)	13.5 (11.3–16.1)	49.1 (45.7–52.6)	
Tennessee	58.3 (54.0–62.4)	37.2 (33.2–41.4)	21.1 (17.7–24.8)	41.7 (37.6–46.0)	
Texas	67.0 (63.6–70.1)	36.9 (33.6–40.3)	30.1 (27.0–33.3)	33.0 (29.9–36.4)	
Vermont	63.1 (59.8–66.3)	39.2 (35.9–42.5)	23.9 (21.2–26.9)	36.9 (33.7–40.2)	
Virginia	63.9 (59.2–68.3)	40.6 (35.9–45.3)	23.3 (19.5–27.6)	36.1 (31.7–40.8)	
Washington	68.1 (64.7–71.2)	35.2 (32.0–38.6)	32.9 (29.7–36.1)	31.9 (28.8–35.3)	
West Virginia	49.0 (45.2–52.8)	32.8 (29.3–36.4)	16.2 (13.6–19.3)	51.0 (47.2–54.8)	
Wisconsin	51.8 (47.6–56.0)	38.7 (34.7–42.9)	13.1 (10.6–16.0)	48.2 (44.0–52.4)	

**Abbreviations:** CI = confidence interval, WIC = Special Supplemental Nutrition Program for Women, Infants, and Children.

* “Any” is the sum of “Rarely or sometimes” and “Often or always.”

^†^ Weighted percentage.

Use of at least one type of soft bedding was reported by 38.5% of respondents, ranging from 28.7% in Illinois to 52.6% in New York City (Table 3). The most frequently reported types of soft bedding were bumper pads (19.1%) and plush or thick blankets (17.5%), followed by pillows (7.1%), infant positioners (6.2%), and stuffed toys (3.1%). Use of at least one type of soft bedding varied by maternal characteristics and breastfeeding at 8 weeks postpartum. The prevalence of soft bedding use was higher among respondents who were Asians/Pacific Islanders or Hispanics compared with members of other race/ethnicity groups, aged <25 years compared with ≥25 years, who had completed ≤12 compared with >12 years of education, who were WIC participants, and who were not breastfeeding at 8 weeks postpartum (Table 3). 

**TABLE 3:** Prevalence of soft bedding* use, by maternal characteristics, gestational age at birth, and breastfeeding at 8 weeks postpartum — Pregnancy Risk Assessment Monitoring System, 13 states and New York City, 2015

Characteristic	Pillows	Blankets	Bumper pads	Toys	Positioner	Any soft bedding*	Chi-square p-value
	% (95% CI)^†^	% (95% CI)^†^	% (95% CI)^†^	% (95% CI)^†^	% (95% CI)^†^	% (95% CI)^†^	
**Total**	7.1 (6.6–7.6)	17.5 (16.8–18.3)	19.1 (18.3–19.9)	3.1 (2.8–3.5)	6.2 (5.7–6.7)	38.5 (37.5–39.5)	—
**Maternal race/ethnicity**							<0.001
White, non-Hispanic	4.3 (3.8–4.9)	14.7 (13.7–15.7)	16.4 (15.4–17.5)	2.5 (2.1–3.0)	5.7 (5.1–6.4)	32.9 (31.6–34.2)	
Black, non-Hispanic	9.9 (8.6–11.5)	22.0 (20.1–24.1)	14.9 (13.2–16.7)	3.8 (2.9–4.9)	7.4 (6.2–8.7)	40.5 (38.2–42.8)	
Hispanic	9.1 (7.7–10.7)	19.3 (17.3–21.4)	35.1 (32.6–37.8)	3.0 (2.2–4.0)	6.2 (5.1–7.5)	52.9 (50.2–55.5)	
Asian or Pacific Islander, non-Hispanic	21.1 (18.0–24.7)	31.1 (27.4–35.0)	18.2 (15.2–21.6)	7.3 (5.4–9.8)	9.5 (7.2–12.4)	54.7 (50.6–58.7)	
American Indian or Alaska Native, non-Hispanic	12.4 (7.3–20.5)	15.1 (9.5–23.0)	12.8 (6.6–23.4)	2.2 (1.3–3.6)	2.8 (1.8–4.5)	35.9 (26.4–46.6)	
**Maternal age group (yrs)**							<0.001
<20	10.9 (8.3–14.1)	27.7 (23.6–32.2)	22.8 (19.0–27.1)	6.4 (4.4–9.2)	7.3 (5.2–10.2)	49.2 (44.6–53.9)	
20–24	9.4 (8.1–10.8)	24.1 (22.1–26.3)	22.0 (20.0–24.1)	4.4 (3.5–5.6)	6.1 (5.1–7.2)	45.9 (43.5–48.2)	
25–34	6.2 (5.6–6.9)	15.3 (14.3–16.2)	18.0 (17.0–19.0)	2.5 (2.1–3.0)	6.1 (5.5–6.8)	35.9 (34.6–37.2)	
≥35	6.1 (5.1–7.4)	14.4 (12.8–16.2)	18.3 (16.5–20.3)	2.4 (1.8–3.3)	6.6 (5.5–7.9)	35.5 (33.2–37.9)	
**Maternal education level (yrs)**							<0.001
<12	12.6 (10.8–14.6)	22.1 (19.7–24.6)	27.9 (25.2–30.7)	4.9 (3.8–6.4)	8.8 (7.3–10.6)	51.0 (48.0–53.9)	
12	8.6 (7.6–9.9)	23.0 (21.2–24.9)	23.3 (21.5–25.2)	3.6 (2.8–4.4)	6.9 (5.9–8.0)	46.9 (44.7–49.1)	
>12	5.4 (4.8–6.0)	14.6 (13.7–15.5)	15.7 (14.8–16.6)	2.6 (2.2–3.0)	5.5 (4.9–6.1)	32.9 (31.7–34.1)	
**WIC participation during pregnancy**							<0.001
No	4.8 (4.3–5.4)	13.4 (12.5–14.4)	15.6 (14.6–16.6)	2.4 (2.0–2.9)	5.6 (5.0–6.2)	31.7 (30.5–33.0)	
Yes	10.0 (9.1–10.9)	22.7 (21.4–24.0)	23.4 (22.0–24.8)	3.9 (3.3–4.6)	7.1 (6.4–8.0)	47.0 (45.5–48.6)	
**Infant gestation (wks)**							0.410
Term (≥37)	7.0 (6.5–7.6)	17.5 (16.7–18.4)	19.3 (18.4–20.2)	3.2 (2.8–3.6)	6.1 (5.6–6.7)	38.6 (37.6–39.7)	
Preterm (<37)	8.0 (6.6–9.7)	17.8 (15.8–20.1)	16.8 (14.8–19.0)	2.4 (1.6–3.5)	7.5 (6.2–9.1)	37.4 (34.8–40.1)	
**Any breastfeeding at 8 wks**							<0.001
No	7.9 (7.0–8.8)	19.8 (18.4–21.2)	22.1 (20.7–23.6)	4.0 (3.4–4.8)	7.4 (6.5–8.3)	42.7 (41.0–44.4)	
Yes	6.6 (6.0–7.3)	16.1 (15.1–17.0)	17.2 (16.3–18.2)	2.6 (2.2–3.0)	5.4 (4.9–6.0)	35.8 (34.6–37.0)	
**State/City**							<0.001
Alaska	13.0 (10.8–15.6)	18.4 (15.8–21.3)	14.4 (12.0–17.2)	2.6 (1.7–3.8)	5.6 (4.1–7.6)	40.6 (37.2–44.2)	
Illinois	5.9 (4.7–7.4)	12.2 (10.4–14.1)	15.6 (13.7–17.8)	1.7 (1.1–2.6)	3.8 (2.9–5.0)	28.7 (26.2–31.3)	
Iowa	5.7 (3.9–8.2)	14.1 (11.1–17.8)	12.4 (9.7–15.7)	1.0 (0.4–2.6)	4.4 (2.9–6.5)	29.0 (25.0–33.3)	
Louisiana	11.6 (9.7–13.8)	16.7 (14.5–19.3)	18.3 (15.9–21.0)	2.9 (2.0–4.1)	11.7 (9.9–13.9)	41.3 (38.2–44.6)	
Maryland	6.1 (4.7–7.9)	19.2 (16.8–21.9)	12.1 (10.1–14.4)	3.5 (2.4–4.9)	6.4 (5.0–8.2)	35.7 (32.7–38.9)	
Michigan	5.4 (4.1–7.2)	13.2 (11.1–15.6)	12.6 (10.5–15.0)	2.0 (1.3–3.2)	4.7 (3.4–6.4)	29.5 (26.6–32.6)	
Missouri	7.3 (5.7–9.3)	19.6 (17.0–22.5)	17.1 (14.7–19.9)	3.0 (2.0–4.5)	5.7 (4.3–7.6)	37.9 (34.7–41.3)	
New Jersey	9.0 (7.4–10.8)	25.2 (22.5–28.0)	28.2 (25.5–31.1)	4.8 (3.7–6.2)	6.0 (4.7–7.6)	51.8 (48.7–54.9)	
New York (outside of New York City)	5.3 (3.7–7.6)	15.7 (12.8–19.1)	20.2 (16.9–23.9)	2.8 (1.7–4.7)	7.1 (5.2–9.6)	38.2 (34.2–42.5)	
New York City	11.4 (9.7–13.3)	24.5 (22.1–27.0)	27.8 (25.3–30.5)	5.2 (4.0–6.7)	7.0 (5.7–8.6)	52.6 (49.7–55.4)	
Pennsylvania	4.8 (3.5–6.5)	15.5 (13.2–18.2)	19.7 (17.0–22.7)	3.8 (2.6–5.4)	5.8 (4.3–7.6)	36.7 (33.4–40.1)	
Tennessee	6.5 (4.7–9.1)	19.7 (16.5–23.4)	20.2 (16.9–23.8)	2.4 (1.4–4.1)	7.9 (5.9–10.6)	41.4 (37.3–45.7)	
West Virginia	6.2 (4.6–8.4)	16.0 (13.4–19.0)	22.2 (19.2–25.6)	3.5 (2.3–5.2)	7.8 (6.0–10.1)	41.5 (37.8–45.3)	
Wyoming	6.8 (4.6–9.9)	20.6 (16.8–25.0)	20.4 (16.6–24.8)	3.4 (2.0–5.9)	8.9 (6.4–12.3)	41.1 (36.2–46.1)	

**Abbreviations:** CI = confidence interval, WIC = Special Supplemental Nutrition Program for Women, Infants, and Children.

* Soft bedding defined as infant being placed to sleep with any of the following items: pillow, thick or plush blanket, bumper pads, stuffed toys, or an infant positioner.

^†^ Weighted percentage.

## Conclusions and Comment

Among all mothers responding, 21.6% reported placing their infant to sleep in a nonsupine position, 61.4% shared their bed with their infant, and 38.5% reported using soft bedding. The noted variation observed in nonsupine sleep positioning by maternal characteristics is similar to several disparities observed in sleep-related death rates (*2**,**3*). Sleep-related infant deaths have been consistently highest among American Indian or Alaska Native followed by non-Hispanic black mothers (*2*) and those who are aged <20 years and have less education (*3*). Unsafe sleep practices were most commonly reported by younger, less educated, and racial/ethnic minority mothers, suggesting priority groups that might need to be reached with clear, culturally appropriate messages.

While most states and subpopulations observed a significant decline over time in nonsupine sleep positioning, these findings highlight the need to implement and evaluate interventions to continue improving safe sleep practices. Evidence-based approaches to increase use of safe sleep practices include developing health messages and educational tools for caregivers and educating health and child care professionals on safe sleep practices (*11*,*12*). For example, a recent randomized controlled trial among postpartum mothers found a 60-day mobile health program significantly improved uptake of safe sleep practices. The mobile health program included sending frequent emails or text messages with short videos related to infant safe sleep practices (*13*). Other strategies include removing known barriers to safe sleep practices (e.g., providing free or reduced cost cribs for families), identifying and addressing cultural and social practices that are unsafe (e.g., by holding safe-sleep baby showers), and implementing legislative and regulatory supports (e.g., requiring SIDS risk reduction training for licensed child care providers) (*11*).

States and health care providers can play an important role in promoting implementation of AAP safe sleep recommendations in a variety of settings. In the Study of Attitudes and Factors Effecting Infant Care, 55% of caregivers reported receiving appropriate advice, 25% received incorrect advice and 20% received no advice on safe sleep practices from health care providers. Caregivers who received appropriate advice were significantly less likely to place their infants to sleep in a nonsupine position than were those who received inappropriate or no advice on safe sleep practices (*7*). In recent years, state public health agencies have worked with partners to implement a variety of efforts to promote safe sleep, including communication campaigns, messaging delivered during WIC program visits and home-visiting programs, policies in facilities and clinics, and hospital-based quality improvement initiatives and collaboratives.^¶¶^ States aiming to improve safe sleep practices can examine successful interventions that have been implemented in other states. For example, the Massachusetts Perinatal-Neonatal Quality Improvement Network implemented a safe sleep initiative in neonatal intensive care units that improved safe sleep practices by modeling safe practices for parents of medically stable premature infants in advance of infant discharge (*14*).*** The Tennessee Department of Health demonstrated that having a hospital policy to correctly model safe sleep practices reduced the percentage of infants placed to sleep in an unsafe environment (e.g., not on their back) while in the hospital by nearly half (*15*). Finally, state participation in national initiatives, such as the National Action Partnership to Promote Safe Sleep Improvement and Innovation Network^†††^ and Collaborative Improvement and Innovation Network to reduce infant mortality,^§§§^ can help facilitate and monitor the use of evidence-based strategies related to safe sleep according to standardized metrics of success.

Continued surveillance of infant sleep practices in the United States is necessary to monitor whether the prevalence of safe sleep practices is improving, especially among populations where sleep-related infant mortality is disproportionately high. The state-specific estimates derived from PRAMS can complement other data sources used to assess initiatives to reduce sleep-related infant deaths. Of note, CDC also supports 16 states and two jurisdictions through its Sudden Unexpected Infant Death (SUID)^¶¶¶^ Case Registry to monitor sleep-related deaths and related circumstances, including the sleep environment. This surveillance effort, which captures 30% of all SUID cases in the United States, focuses on improving data quality and completeness of SUID investigations to inform strategies to reduce sleep-related deaths (*16*).****

The findings in this report are subject to at least three limitations. First, results are limited to states that implemented PRAMS, met the required response rate threshold for inclusion in data analysis, and included questions regarding safe sleep practices on their state-specific PRAMS survey. Second, AAP recommends placing the infant to sleep in the supine position every time; however, the PRAMS survey only asked respondents the sleep position their infant was placed most often. Also, prior to 2016, PRAMS collected data on the unsafe practice of bed sharing, but not on the AAP-recommended practice of room sharing. Finally, PRAMS data are self-reported and might be subject to both recall and social desirability biases.

Despite recommendations from AAP regarding safe sleep practices for infants, this report demonstrates that placement of infants in a nonsupine sleep position, bed sharing with infants, and use of soft bedding are commonly reported by mothers. Evidence-based interventions that encourage infant safe sleep practices by caregivers, particularly within populations where unsafe infant sleep practices are higher, could help reduce sleep-related infant mortality.

**Key Points**

• Infant safe sleep practices recommended by the American Academy of Pediatrics (AAP), including placing infants to sleep on their backs, room sharing but not bed sharing, and keeping soft objects and loose bedding out of the infant’s sleep environment, can help reduce sleep-related infant deaths; however, implementation of these recommendations remains suboptimal.

• Approximately one in five mothers reported placing their infant to sleep on their side or stomach. More than one half reported bed sharing with their infant, and more than one third reported using soft bedding in the infant’s sleep environment. Unsafe sleep practices varied by state, race/ethnicity, age, education, and participation in the Special Supplemental Nutrition Program for Women, Infants, and Children.

• Health care providers and state-based and community-based programs can identify barriers to safe sleep practices and provide culturally appropriate counseling and messaging to improve infant safe sleep practices.

• Additional information is available at https://www.cdc.gov/vitalsigns/.

## Footnotes

* A small percentage of respondents (<4%) selected more than one sleep position. Respondents selecting multiple positions were classified as placing their infant in a nonsupine sleep position. Denominator includes supine, on stomach, on side only, and combinations of any of the three positions.

† The 32 states include Alabama, Alaska, Arkansas, Colorado, Connecticut, Delaware, Hawaii, Illinois, Iowa, Louisiana, Maryland, Massachusetts, Michigan, Missouri, Nebraska, New Hampshire, New Jersey, New Mexico, New York (excluding New York City), Ohio, Oklahoma, Oregon, Pennsylvania, Tennessee, Texas, Utah, Vermont, Virginia, Washington, West Virginia, Wisconsin, and Wyoming.

§ The 15 states include Delaware, Hawaii, Illinois, Maryland, Massachusetts, Missouri, Nebraska, New Jersey, Oklahoma, Pennsylvania, Utah, Vermont, Washington, West Virginia, and Wyoming.

¶ The 14 states include Alaska, Connecticut, Delaware, Louisiana, Nebraska, New Jersey, Pennsylvania, Tennessee, Texas, Vermont, Virginia, Washington, West Virginia, and Wisconsin.

** The 13 states include Alaska, Illinois, Iowa, Louisiana, Maryland, Michigan, Missouri, New Jersey, New York (excluding New York City), Pennsylvania, Tennessee, West Virginia, and Wyoming.

†† To provide general guidance on the statistical differences, 95% CIs for the prevalence were compared across groups, with an emphasis on identifying differences (i.e., nonoverlap of CIs) between categories within the selected variables. This typically conservative approach might fail to note differences between estimates more often than formal statistical testing. Overlap between confidence intervals does not necessarily mean there is no statistical difference between estimates.

§§ Alaska Native information available for Alaska only.

¶¶
https://www.cdc.gov/reproductivehealth/maternalinfanthealth/pqc.htm .

***
http://www.mapnqin.org/neonatal-projects/

†††
http://www.nichq.org/project/national-action-partnership-promote-safe-sleep-improvement-and-innovation-network-nappss .

§§§
https://mchb.hrsa.gov/maternal-child-health-initiatives/collaborative-improvement-innovation-networks-coiins .

¶¶¶ Sudden unexpected infant death (SUID) is the death of an infant aged <1 year that occurs suddenly and unexpectedly, and whose cause of death is not immediately obvious before investigation.

****
https://www.cdc.gov/sids/CaseRegistry.htm .
